# A Resonant Pressure Microsensor Based on Double-Ended Tuning Fork and Electrostatic Excitation/Piezoresistive Detection

**DOI:** 10.3390/s18082494

**Published:** 2018-08-01

**Authors:** Xiaoqing Shi, Yulan Lu, Bo Xie, Yadong Li, Junbo Wang, Deyong Chen, Jian Chen

**Affiliations:** 1 State Key Laboratory of Transducer Technology, Institute of Electronics, Chinese Academy of Sciences, Beijing 100190, China; shixiaoqing16@mails.ucas.ac.cn (X.S.); luyulan15@mails.ucas.ac.cn (Y.L.); xiebo11@mails.ucas.ac.cn (B.X.); liyadong16@mails.ucas.ac.cn (Y.L.); chenjian@mail.ie.ac.cn (J.C.); 2 University of Chinese Academy of Sciences, Beijing 100049, China

**Keywords:** resonant pressure microsensor, electrostatic excitation/piezoresistive detection, double-ended tuning forks, MEMS

## Abstract

This paper presents a resonant pressure microsensor relying on electrostatic excitation and piezoresistive detection where two double-ended tuning forks were used as resonators, enabling differential outputs. Pressure under measurement caused the deformation of the pressure sensitive membrane, leading to stress buildup of the resonator under electrostatic excitation with a corresponding shift of the resonant frequency detected piezoresistively. The proposed microsensor was fabricated by simplified SOI-MEMS technologies and characterized by both open-loop and closed-loop circuits, producing a quality factor higher than 10,000, a sensitivity of 79.44 Hz/kPa and an accuracy rate of over 0.01% F.S. In comparison to the previously reported resonant piezoresistive sensors, the proposed device used single-crystal silicon as piezoresistors, which was featured with low DC biased voltages, simple sensing structures and fabrication steps. In addition, the two double-ended tuning forks were used as resonators, producing high quality factors and differential outputs, which further improved the sensor performances.

## 1. Introduction

Nowadays, micro pressure sensors are widely used in the fields of aerospace and meteorology [1]. There are several common types of pressure sensors, such as capacitive pressure sensors [2,3], piezoresistive pressure sensors [4,5], piezoelectric pressure sensors [6,7] and resonant pressure sensors [8,9]. In comparison to other pressure sensors, resonant pressure sensors are featured with “quasi-digital” outputs, high resolutions, and long-term stabilities due to the quantification of the variations of intrinsic resonant frequencies [10].

Depending on different excitation and detection mechanisms, resonant pressure sensors can be classified into electrothermal excitation and piezoresistive detection [11,12], electromagnetic excitation and electromagnetic induction detection [13,14,15], electrostatic excitation and piezoresistive detection [16,17] electrostatic excitation and capacitive detection [18,19,20].

Among the different excitation mechanisms, in electrothermal excitations, alternative currents are applied on resistors to produce cyclic thermal stresses to vibrate the resonant beams. This excitation mechanism is not favorable due to its high energy consumption. In electromagnetic excitations, alternative currents are applied on the resonant beams, which then vibrate due to the induced ampere forces resulting from surrounding electromagnetic fields. Since magnets are always required in this excitation mode, they lead to enlarged device volumes and pose potential threats for aerospace applications. Electrostatic excitations apply cyclic voltages on two electrode plates, and the produced electrostatic forces are used to vibrate the resonant beams. In comparison to the other types of excitations, this approach is featured with low energy consumption and small device volumes. It should be noted that although there is a squeeze film air damping between two parallel electrodes, high quality factors can be maintained in the environments of high vacuums [21].

Among the different detection mechanisms, capacitive detections are susceptible to interferences of current noises due to high impedance and parasitic capacitances, which simultaneously suffer from the problem of weak signals. In comparison, piezoresistive detections have low impedance, where current noises and low-frequency noises can be effectively reduced. Although this detection approach is prone to variations of environmental temperatures, differential setups with specific algorithms of temperature compensations can sufficiently address this issue [22].

Thus, this study presents a resonant pressure sensor relying on electrostatic excitation and piezoresistive detection where differential resonators based on double-ended tuning forks are used. Compared with the previously reported devices based on the same excitation and detection schemes, the proposed resonant pressure sensor is featured with (1) a new driving and a sensing structure, and (2) a differential setup to decrease the side effect of temperature. In addition, the fabrication process which includes photolithography, deep reactive ion etching (DRIE) and silicon-glass anodic bonding is significantly less complicated than the previously reported counterparts [16] which needed dense boron etch stop, wafer thinning and fusion bonding. The proposed microsensor was characterized in both open-loop and closed-loop manners, producing a quality factor higher than 10,000, a differential sensitivity of 79.44 Hz/kPa and an accuracy rate of greater than 0.01% F.S.

## 2. Design and Simulation

**Figure 1:**
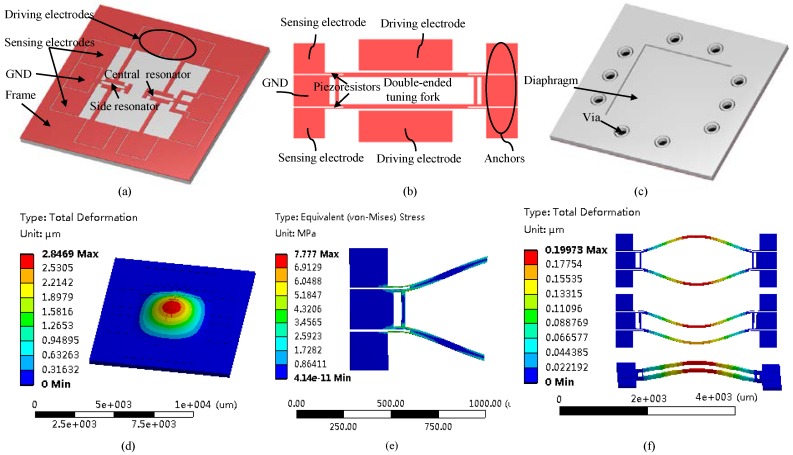
Schematic and working principle of the resonant pressure microsensor relying on electrostatic excitation and piezoresistive detection (**a**) where double-ended tuning forks (**b**) were used as resonators. In addition, the through-silicon vias surrounding the pressure sensitive membrane (**c**) were adopted in annular shapes for electrical isolation. Pressures under measurement cause the deformations of the pressure sensitive membrane (**d**) leading to stress buildups of the resonators (**e**) with corresponding shift of the resonant frequency. The first three resonant modes of the microsensor were illustrated in (**f**) with corresponding resonant frequencies of 89.4 kHz, 89.8 kHz and 121.1 kHz respectively. Note that (1) a differential design was adopted in this study, which can further increase the sensitivity and linearity of the pressure sensor and (2) the results of numerical simulations for (**d**–**f**) were not to scale.

Figure 1a–c shows the overall structure of the resonant pressure sensor, which mainly consists of a pressure sensitive membrane and two single crystal silicon resonators based on double-ended tuning forks. In each resonator, it is excited into a dynamically balanced mode of oscillation by the electrostatic forces produced by parallel electroplates and detected based on the piezoresistive resistors positioned at the end of the forks. Two resonators were deployed on the central and side areas of the membrane respectively, which are named as “central resonator” and “side resonator”.

Figure 1d–f shows the working principle of the resonant pressure sensor. When the pressure sensitive membrane senses the pressure variations, it produces deformation, thus stressing the resonator and modulating the intrinsic resonant frequencies of the resonators, which are further detected by piezoresistive resistors. In this study, a differential setup was used and the pressure under measurement led to an increase in the resonant frequency of the central resonator and at the same time a decrease in the resonant frequency of the side resonator.

In designing this resonant pressure sensor, there are several considerations. The functions of the pressure sensitive membrane are to translate outside pressures to intrinsic frequencies of fork resonators. A decrease in the membrane thickness can enhance the device sensitivities and at the same time produce a lower resonant frequency of the membrane, which should be significantly lower than the resonant frequencies of fork resonators. However, further decrease in the membrane thickness can decrease the detection range of the pressure sensors. Thus, in this study, a geometry of 4.8 mm × 4.8 mm × 120 µm was chosen for the pressure sensitive membrane.

The vias surrounding the pressure sensitive membrane were used to form electrical connections with the outside circuit. An annular shape was adopted to avoid the adhesions between the substrate and the electrodes sputtered within the vias, which can significantly increase the yield of the sensor.

In addition, the geometry and the positions of the fork resonators, driving electrodes and piezoresistors can also affect the performance of the proposed sensor. More specifically, the geometrical parameters of the fork resonators can affect intrinsic resonating frequencies and detection sensitivities. In this study, the resonant beams with dimensions of 1200 µm × 20 µm × 40 µm were used, which can produce a fundamental frequency of ~88 kHz, significantly higher than 70 kHz, the resonant frequency of the pressure sensitive membrane to avoid mutual interferences. In order to match the sensitivities of the resonators for differential outputs, the central resonator was positioned about 920 µm on the right side from the center of the pressure sensitive membrane within the region of positive stress, with an increase in the intrinsic frequency in response to external pressures. On the other hand, the side resonator was positioned about 1680 µm on the left side from the center of the pressure sensitive membrane within the region of positive stress, demonstrating a corresponding decrease in the resonant frequency in response to external pressures (see Figure 1).

In addressing the driving electrodes, the gap should be as small as possible to maximize the driving force. When the feasibility of fabrication was taken into consideration, a gap of 4 µm with a height of 40 µm was used in this study.

In detecting the resonant vibrations, the position and geometrical information of piezoresistors were two key factors. The stress produced at the upper and lower surfaces, rather than the medium region at the end of the fork resonators, were maximal and thus, piezoresistors were positioned in these locations, which could also be easily integrated with detection electrodes. The widths of the piezoresistors were suggested to be as small as possible to sense the produced stress, which could enlarge the changes of the piezoresistance.

In addition, there are three vibration modes shown in Figure 1f, which are the first, second and third modes respectively. Among these three modes, the first two modes are in-plane modes with close frequencies and the third is the out-of-plane mode with a significantly higher frequency which is beyond the scope of this article. Each resonator is excited to vibrate in a lateral in-phase mode (the first mode), which can minimize the mechanical coupling between the resonators and the pressure sensitive membrane. The second in-phase mode can be avoided by adjusting the driving voltages. Furthermore, the vibrating directions of the resonators and the pressure sensitive membrane are perpendicular, which further reduces the energy coupling between them.

## 3. Fabrication

**Figure 2:**
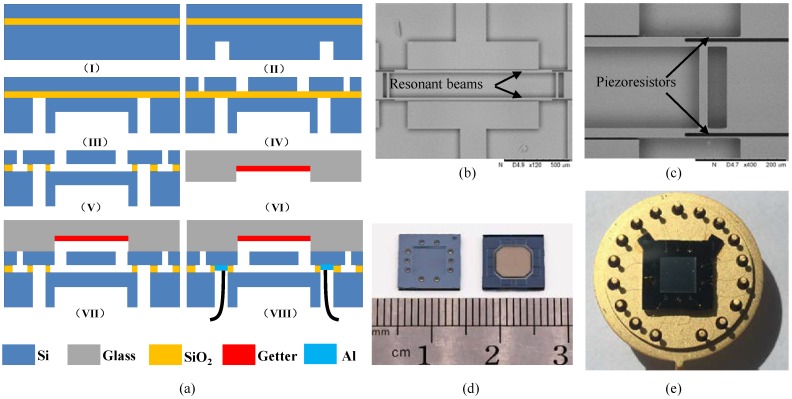
(**a**) The fabrication process of the resonant pressure microsensor: (**I**) thoroughly clean the SOI wafer, (**II**) and (**III**) form the pressure sensitive membrane and the holes for electrical connections, (**IV**) pattern the device layer, (**V**) release the oxide layer beneath the resonators, (**VI**) deposit a layer of getter material on glass covers, (**VII**) conduct silicon-to-glass anodic bonding, (**VIII**) form electrical connections. (**b**,**c**) SEM pictures of fabricated resonators based on double-ended tuning forks. (**d**,**e**) The pictures of the fabricated sensors before and after forming electrical connections with the surrounding circuits.

The resonant pressure sensors were fabricated based on a 4-inch SOI wafer (a (100) *n*-type substrate layer of 300 µm, an oxide layer of 2 µm, a <110> *p*-type device layer of 40 µm with a conductivity of 0.01 S/m) by conventional microfabrications, which mainly include photolithography, deep reactive ion etching (DRIE), and silicon-glass anodic bonding (see Figure 2a). In the first step, the SOI wafer was thoroughly cleaned based on conventional procedures in the IC industry (see Figure 2(aI). Next, DRIE was used to form the pressure sensitive membrane and through-silicon vias (see Figure 2(aII,III). Then, DRIE was used to form the layer of the resonators, including the resonant beams, excitation electrodes and detection piezoresistors (see Figure 2(aIV), which were further released by removing the underneath oxide layer by gaseous hydrofluoric acid (HF) (see Figure 2(aV). In the next step, glass covers were formed by HF etching, deposited with getter materials (see Figure 2(aVI) and anodically bonded to the patterned SOI wafer to form a sealed vacuum chamber (see Figure 2(aVII). In the end, Al was sputtered on the backside to form electrical connections through vias with the surrounding world (see Figure 2(aVIII).

Figure 2b,c shows the SEM pictures of a fabricated resonator with piezoresistors highlighted, confirming the feasibility of the proposed fabrication processes. In addition, Figure 2d,e) show the pictures of the developed sensor with and without packaging, suggesting that the dimensions of the sensor unit were 9 mm × 9 mm and a diameter of 22 mm before and after packaging.

## 4. Results and Discussion

**Figure 3:**
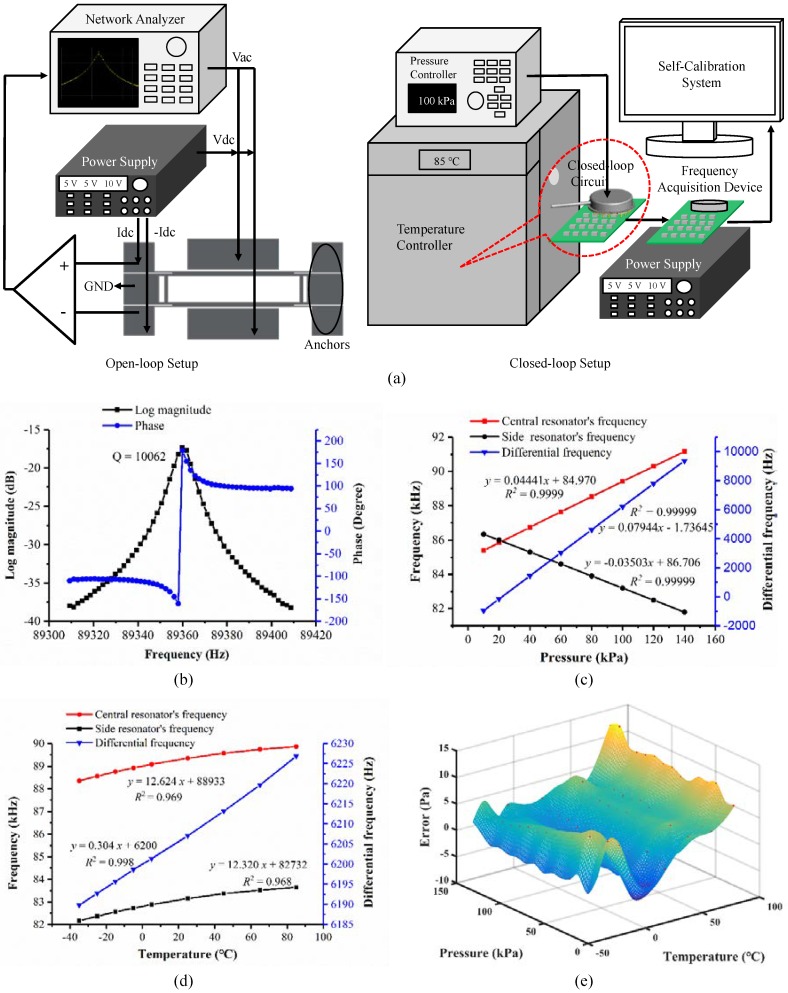
(**a**) Schematic of the experimental setup for both open-loop and closed-loop measurements of the proposed resonant pressure sensors. (**b**) Open-loop testing results where the quality factor and the phase of the developed resonant pressure microsensor were obtained. (**c**) Closed-loop testing results where the resonant frequency of the central resonator (f1) and side resonator (f2) and the differential output (f1–f2) of the developed resonant pressure microsensor versus applied pressure at 25 °C. (**d**) The shift of resonant frequencies as a function of temperature in the range of −35 °C to 85 °C under a predefined pressure of 100 kPa. (**e**) The errors of the developed resonant pressure microsensors in a pressure range of 10 kPa to 150 kPa and a temperature range of −35 °C to 85 °C. The quantified errors were within ±14 Pa, within 0.01% F.S.

The fundamental frequency and quality factor (Q-factor) of the proposed resonant pressure sensors were first quantified using an E5601B Network Analyzer (Agilent, Santa Clara, CA, USA) in an open-loop manner. Pressures were applied to the pressure sensitive membrane with a FLUKE PPC4 pressure controller and temperature was controlled by a temperature controller (SU-262, ESPEC) in a temperature range of −35 °C–85 °C (temperature intervals of 10 °C and 20 °C in the negative and positive temperature regions, respectively) (see Figure 3a). As shown in Figure 3b, the fundamental frequency of the central resonator was 89.36 kHz under 100 kPa in normal temperature and the phase was ~180 degree. The quantified Q-factor was higher than 10,000, indicating that the proposed pressure sensors functioned in the environment of high vacuum. These results further confirm the effectiveness of the anodic bonding used in this study.

Then, closed-loop characterization of the proposed pressure sensor was conducted using a home-developed self-oscillating circuit providing a 90 degree phase shift and an automatic gain control [13] (see Figure 3a). More specifically, both the direct current voltage (Vdc) of +10 V and the alternative current voltage (Vac) generated by the self-oscillating circuit were applied to electrode plates, producing vibrations of resonators. In the sensing electrodes, +0.5 mA and −0.5 mA were applied based on two direct current sources to enable differential outputs. The sensing electrodes can also output the direct and alternative voltages when the resonators vibrate resonantly. Then, the direct voltage was inhibited and the alternative voltage was amplified to drive the resonators.

Figure 3c shows the resonant frequency of the pressure sensor as a function of applied pressure at 25 °C. The resonant frequency of the central resonator was observed to increase along with the applied pressure, while the intrinsic frequency of the side resonator decreased along with the applied pressure. The sensitivities of the two resonators were about 44.41 Hz/kPa and 35.03 Hz/kPa, respectively, within the pressure range of 10 to 150 kPa at 25 °C. The differential sensitivity of the proposed pressure sensor was quantified as 79.44 Hz/kPa with a linear coefficient of 0.99999, indicating a linear response of the developed pressure sensor.

Figure 3d shows the shift of the resonant frequency as a function of the temperature range of −35 °C to 85 °C under the pressure of 100 kPa. More specifically, the temperature sensitivities of the central and side resonators were quantified as 12.62 Hz/°C and 12.32 Hz/°C, respectively. The temperature sensitivity of the differential setup was quantified as 0.30 Hz/°C, indicating that the differential structure proposed in this study can reduce the side effects of the temperature to an extent.

The developed pressure sensor with the closed-loop circuit was calibrated by a self-calibration system under a group of pressures and compensated by a polynomial fitting [13,22]. Figure 3e shows the errors between the fitting and the real pressure values within the pressure range of 10 kPa to 150 kPa and the temperature range of −35 °C to 85 °C. It was observed that the errors of the developed pressure sensor were within ±14 Pa, and the accuracy of the pressure sensor was better than 0.01% F.S.

## 5. Conclusions

A new resonating structure with double-ended tuning forks and electrostatic excitation/piezoresistive detection was presented where the double resonators which were positioned on the central and side locations of pressure sensitive membranes formed differential modes to decrease the side effects of the temperature on sensor performances. The proposed resonant pressure sensors were fabricated by conventional SOI-MEMS technologies, and characterized in both open-loop and closed-loop manners, producing quality factors higher than 10,000, sensitivities of 79.44 Hz/kPa and accuracy rates of higher than 0.01% F.S. Compared to previously reported counterparts, in this study, single-crystal silicon based piezoresistors were used, which were featured with low DC biased voltages, simple sensing structures and fabrication steps. Future developments may focus on (1) technical improvements in further improving quality factors and temperature working ranges and (2) field applications in the atmospheric data computers of airplanes.
